# Molecular identification and wing variations among malaria vectors in Akure North Local Government Area, Nigeria

**DOI:** 10.1038/s41598-022-11917-y

**Published:** 2022-05-10

**Authors:** Adebayo Victor Akeju, Titus Adeniyi Olusi, Iyabo Adepeju Simon-Oke

**Affiliations:** Parasitology and Public Health Unit, Department of Biology, Federal University of Technology Akure, Akure, Nigeria

**Keywords:** Molecular biology, Zoology

## Abstract

Members of the *Anopheles gambiae* complex and *Anopheles* *funestus* group are significant vectors of the malaria parasite *Plasmodium* species in the Afro-tropical region of the world. Molecular identification and variation in the wing were studied among female *An.* *Gambiae* complex and *An. funestus* group, to investigate morphological variations in the wing of local vectors populations of adult female mosquitoes found in five different locations in Akure North Local Government Area of Ondo State (Oba—Ile, Igoba, Isinigbo, Ita—Ogbolu and Iju). The variations in the wing character were found in the 3rd main dark spot area (Pre-apical dark spot—character 8) on the coastal region (Vein region I) of *Anopheles gambiae* complex wing; with two types (A and B) of wings identified with *An. gambiae* complex in the study area. Molecular study shows that all the wing type A are *Anopheles gambiae* s.s., they represent 53.39% of the total *An. gambiae* complex in the study area. Some of the *Anopheles gambiae* s.s. (28.30%) and all *An. arabiensis* (18.30%) were found with wing type B. Among 750 individual *Anopheles* mosquito species identified using Polymerase Chain Reaction (PCR method), 433 samples representing 57.73% were *An. gambiae* s.s. while 97 (12.93%) samples were *An. arabiensis*. *Anopheles leesoni* was the only member of *the An. funestus* group identified in the study area. *Anopheles leesoni* mosquitoes identified in the study location were 182, representing 24.27% of the total *Anopheles* mosquito species identified using the molecular method. *Anopheles gambiae* s.s., *An*. *arabiensis*, and *An. leesoni* are only *Anopheles* mosquito species responsible for malaria transmission in the study area. *Anopheles leesoni* was the only member of *the An. funestus* group identified in the study area.

## Introduction

Malaria is a severe disease condition in Africa and Asia continents caused by protozoa of the genus *Plasmodium*. The malaria vector belongs to the genus *Anopheles*. They are the well-studied among the subfamily Anophelinae due to their significant role in transmitting major parasitic pathogens of public health importance^1,2^. Malaria infection is a significant public health problem in Africa. In Africa, the primary vector of malaria parasites belongs to *Anopheles gambiae* complex and *Anopheles funestus* group^3–5^. *Anopheles gambiae* complex contains eight members with similar morphological features, especially among the closest sibling species *Anopheles gambiae* s.s., *Anopheles arabiensis*, and *Anopheles coluzzii*; the primary vector of malaria parasites in Africa^5^. Among the member of *the Anopheles funestus* group, *Anopheles funestus* s.s. has been reported as an important vector of malaria parasites in the Afro-tropical region. Another member of *the Anopheles funestus* group, *Anopheles leesoni*, whose source of blood meal is animal, has been reported predominating in human dwelling and feeding on human blood^6,7^. The existence of species complexes in *Anopheles* vectors has led to difficulties in precisely identifying sibling species (isomorphic species) and subspecies (morphologically, cytologically, and polymorphic races) members that possess identical morphology or minimal morphological distinction^8,9^. The modern-day taxonomy seems to have solved the problems associated with complexes species known to be groups of morphologically indistinguishable species that are genetically different and differ significantly in vectorial potential. There is limitation in the uses of morphological approaches in *Anopheles* species identification. This could cause discrepancy and result in identification problem^10^. The number of sensilla coeloconica, the value of the papal index, and the eggs' shape can differentiate *Anopheles* species complexes. Though, none of these characters gave complete discriminant as the morphological characters of adults are variable and overlap in many instances. Identifying vectors that belong to species complexes has long been a stumbling block in malaria epidemiology and control^11^. Thus, this research aims to identify the vector of malaria parasites using molecular techniques and variation in morphological characters of the wing in five major settlements in Akure North Local Government Area of Ondo State, Nigeria.

## Methods

### Study area

This research was carried out in Akure North Local Government Area (LGA) of Ondo State, Nigeria. The study site and environmental factors associated with Anopheles mosquitoes has been described earlier by Olusi et al.^12^.

### Collection and rearing of *Anopheles* mosquitoes

*Anopheles gambiae* and *An. funestus* mosquitos’ larvae were collected randomly from different locations in five selected communities, which include Oba—Ile, Igoba, Isinigbo, Ita—Ogbolu and Iju. The larvae were identified by their characteristic horizontal positioning on the water surface and carefully collected into plastic containers. Each of the containers were well labelled. The containers were loosely capped to avoid suffocation and immediately transported to the insectary and reared to adult stage at Parasitology and Public Health Research Laboratory, Federal University of Technology, Akure.

### Morphological study of the adult female *Anopheles* mosquito wing

Upon adult emergence, *Anopheles* mosquitoes were identified using standard morphological characters keys supplied in Gunathilaka^10^, Nagpal and Sharma^13^. Species identification was done based on taxonomic keys^14^. The wing characters of Afrotropical *Anopheles* mosquitoes responsible for transmitting malaria parasites was considered following the morphological characters and wing spots nomenclature provided by Gillies and Coetzee^14^.

### DNA extraction

Before the DNA extraction, the individual mosquito samples were store in 70% ethanol and refrigerated. The genomic deoxyribonucleic acid (DNA) was extracted from the thorax and abdomen tissues of individual mosquitoes using genomic DNA purification kit for animal tissue provided by Jena Bioscience.

### Amplification of extracted DNA

The amplification of extracted DNA was carried out following the protocol provided by Solis BioDyne for a Polymerase Chain Reaction (PCR). The premixed ready to load master mix containing 4.0 μl pre-mix, 5.25 μl ddH_2_O, 0.5 μl universal primer of *Anopheles gambiae* complex (5-GTG TGC CCC TTC CTC GAT GT-3) at concentration of 0.3 μM for forward reaction, 0.5 μl species-specific primer of *An. arabiensis* (AR) 5-AAG TGT CCT TCT CCA TCC TA-3, *An. gambiae* s. s. (GA) 5-CTG GTT TGG TCG GCA CGT TT-3, *An. merus* (ME) 5-TGA CCA ACC CAC TCC CTT GA-3, and 0.25 µl species-specific primer of *An. quadriannulatus* (QD) 5-CAG ACC AAG ATG GTT AGT AT-3 at concentration of 0.3 μM for the reverse reaction^15^. The prepared PCR master mix of 12.5 μl was added into each 200 μl tube, after which 1.0 μl of extracted DNA was added to each of the tubes. Each tube was loaded into the PCR machine, and an appropriate program was selected. The PCR product was allowed through the first PCR cycle for initial denaturation at 95 °C for 2 min, after which it underwent denaturation at 95 °C for 30 s. The products were annealing at 55 °C for 30 s, followed by product extension at 72 °C for 40 s.

Appropriate program for *Anopheles funestus* amplification process was selected following the protocol described by Solis BioDyne for a Polymerase Chain Reaction. The DNA cocktail used for species identification of *Anopheles funestus* group contains 4.0 μl pre-mix, 6.6 μl ddH_2_O, 0.15 µl universal primer (5-TCT GAA CTG CAG GAC ACA T-3); for *An*. *funestus* group, 0.15 µl of species-specific primer of *An. funestus* s. s. (FUN): 5-GCA TCG ATG GGT TAA TCA TG-3, *An. vaneedeni* (VAN): 5-TGT CGA CCT GGT AGC CGA AC-3, *An. rivulorum* (RIV): 5-CAA GCC GTT CGA CCC TGA TT-3, *An. parensis* (PAR): 5-TGC GGT CCC AAG CTA GGT TC-3, *An. leesoni* (LEES): 5-TAC ACG GGC GCC ATG TAG TT-3-3 at concentration of 0.3 μM for the reverse reaction. The prepared master mix of 12.5 μl was added into microcentrifuge tube containing 1.0 μl of extracted DNA template. Polymerase chain reaction product was allowed to pass through initial denaturation at 95 °C for 2 min. The product later went through second denaturation at 95℃ for 30 s. The product was annealing at 45 °C for 30 s, after which extension of the product was done at 72 °C for 5 min.

### Gel electrophoresis of the PCR product

The PCR products were electrophoresed on a 1.5% agarose gel with Tris-Borate Ethylene-diamino tetra acid (TBE) buffer containing 0.89 M Tris-Borate (TB) and 0.02 M Ethylene-di amine tetra acetic acid (EDTA). The gel was stained with 10 μl Ethidium Bromide (EtBr) to detect the presence of amplified DNA fragments. The PCR products were loaded into each well and run at 100 V with not more than 120–150 mA. The gel pictures were taken under ultraviolet light using a gel documentation machine.

### Data analysis and interpretation

The bands that appear on the gel were documented and scored according to *Anopheles* specific species base pairs. Data were subjected to statistical analysis using SPSS Version 26. The population of *Anophele*s mosquitoes was compared among the study locations using Chi-square analysis.

## Results

### Molecular identification of *Anopheles gambiae* complex

**Figure 1:**
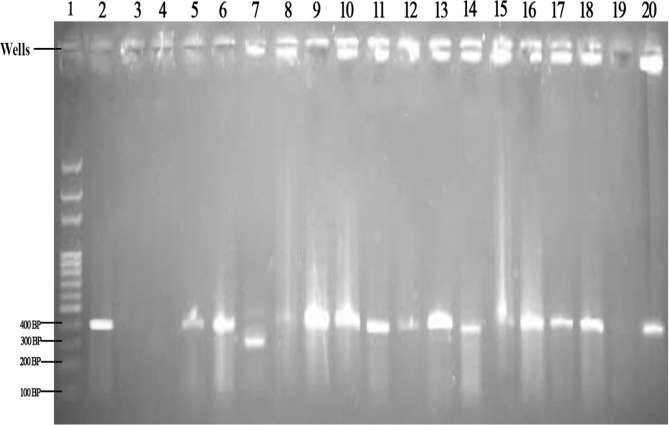
Agarose gel of the DNA fragment of amplified PCR of *Anopheles gambiae* complex. Well 1 represent 100 base pair (BP) DNA ladder. Well 2 a positive control, well 3 a negative control, well 5, 8, 9, 10, 11, 12, 13, 14, 15, 16, 17, 18 and 20 represent *Anopheles gambiae* s. s. 390 base pair. well 7 represent *Anopheles arabiensis* 315 base pair.

Out of 550 morphological identified *Anopheles gambiae* complex, 530 individual *Anopheles gambiae* which represent 96.36% were positive while 20 (3.64%) of the collected samples were negative (Table 1). The main specific *Anopheles gambiae* complex species that was present in the identified samples were *Anopheles gambiae* s. s. and *Anopheles arabiensis*. Molecular analysis confirmed that 78.27% were *Anopheles gambiae* s.s., the DNA bands of this species was molecularly identified on the gel image with 390 base pair. About 17.64% of samples were confirmed to be *Anopheles arabiensis*, this was identified with 315 base pair on the gel image (Fig. 1).

**Table 1:** Distribution of molecular identified *Anopheles gambiae* complex in the study area.

Location	No examined	*Anopheles gambiae* s. s. (%)	*Anopheles arabiensis* (%)	Negative (%)
Igoba	110	81 (73.64)	26 (23.64)	3 (2.73)
Isinigbo	110	83 (75.46)	23 (20.91)	4 (3.64)
Oba–Ile	110	98 (89.90)	10 (9.09)	2 (1.82)
Iju	110	87 (79.09)	18 (16.36)	5 (4.55)
Ita–Ogbolu	110	84 (76.36)	20 (18.18)	6 (5.46)
Total	550	433 (78.27)	97 (17.64)	20 (3.64)

Chi-square (χ^2^) = 12.18; df = 4; P = 0.14 (P > 0.05).

Both identified species were well distributed across the five study locations. The highest number of *Anopheles gambiae* s.s. was found among the mosquito samples of *Anopheles gambiae* complex collected from Oba–Ile with 89.90%, while the least number of *Anopheles gambiae* s. s. was recorded from Igoba (73.64%) and Isinigbo (75.46%). The number of *Anopheles arabiensis* mosquito from these five locations were small compare to *Anopheles gambiae* s. s., the percentage of *Anopheles arabiensis* molecularly identified from Igoba, Isinigbo and Ita-ogbolu were 23.64%, 20.91% and 18.18% respectively. The least were from Oba–Ile (9.09%). There was significant difference (Chi-square = 12.18; df = 4; P > 0.05) between these two species of *Anopheles gambiae* complex present in the study area.

### Variation in *Anopheles gambiae* complex wing types

**Figure 2:**
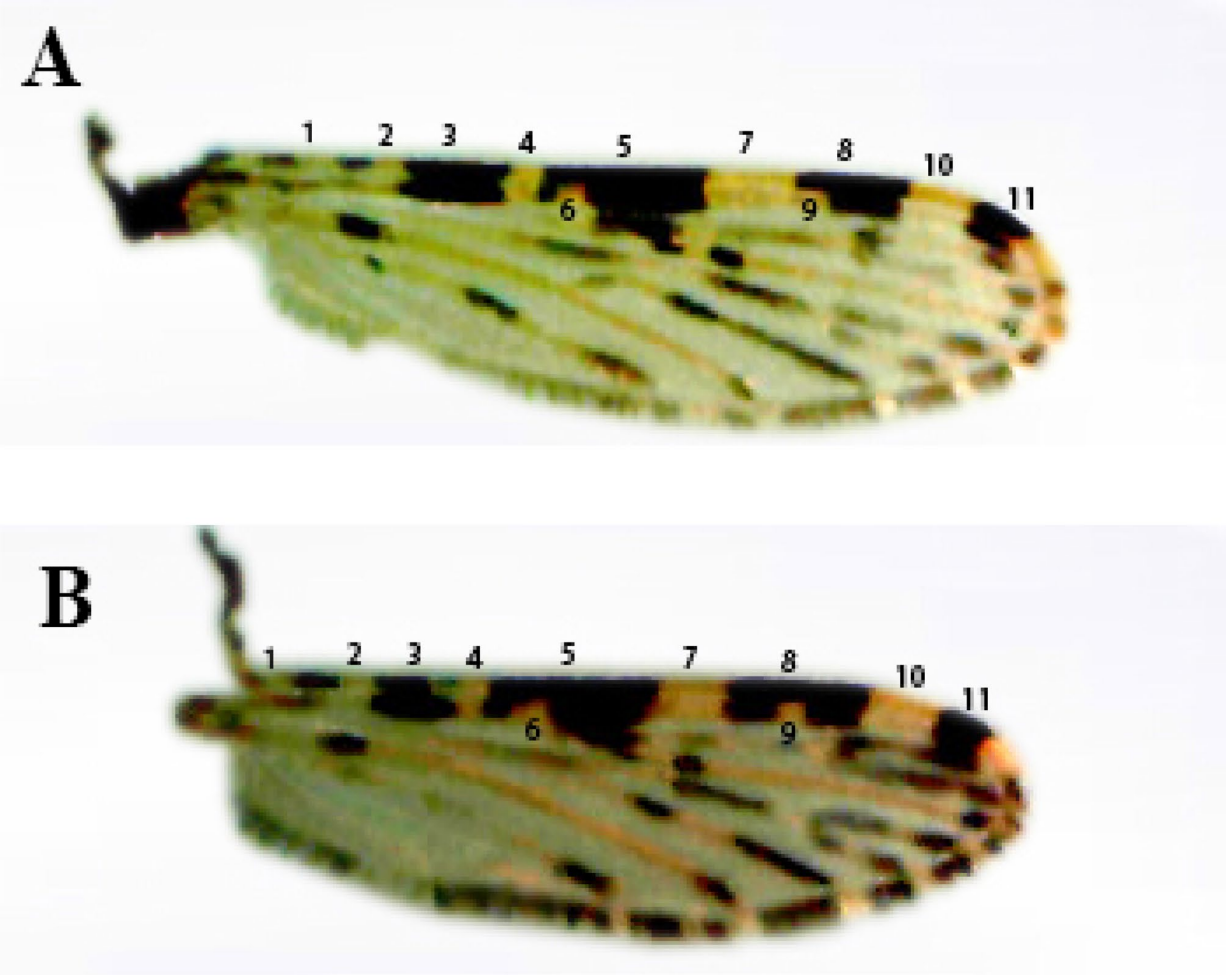
(**A**) Morphological appearance and nomenclature of female *Anopheles gambiae* s. l., wing type A: wing with interrupted pale spot (Character 9) fused with proceeding pale spot of the sub-coastal pale spot. (**B**) Morphological appearance and nomenclature of female *Anopheles gambiae* s. l., wing type B: wing with a pale interruption found within the pre-apical dark spot (character 8). Wing nomenclature: (1) Humoral pale spot. (2) Pre-sector pale spot, (3) Pre-sector dark spot, (4) Sector pale spot, (5) Median dark spot, (6) Accessory sector pale spot, (7) Sub-coastal pale spot, (8) Pre-apical dark spot, (9) Interruption of 3rd main dark area, (10) Sub-apical dark spot, (11) Apical pale spot.

The two distinctive wing types of *Anopheles gambiae* complex collected from the study area were studied, this was done alongside with the molecular confirmation of the specific species. These two types of the wing were presented earlier on Fig. 2A and B. The variations in the wing character were found in the 3^rd^ main dark spot area (Pre-apical dark spot—character 8) on coastal region (Vein region I). The most common type of the wing in the population of *An. gambia*e complex studied were those with the interrupted pale spot (Character 9; Fig. 2A) fused with proceeding pale spot of the sub-coastal pale spot. In the other type, pale interruption was found within the pre-apical dark spot (Character 8; Fig. 2B). The result of the molecular analysis confirmed that all the samples with wings having a pale interruption proceed with the sub-coastal pale spot (wing type A) are *An. gambiae* s. s, which represented 53.39% of the total number of positive samples for *An. gambiae* complex. Among the wing type B (those with pale interruption found within the pre-apical dark spot), 60.73% of the samples was *Anopheles gambiae* s. s., this represented 28.30% of the total number of positive samples for *An. gambiae* complex while 39.27% were *Anopheles arabiensis*/this represented 18.30% of total number of positive samples for *An. gambiae* complex. There was significant difference (Chi-square = 24.39; df = 4; P < 0.05) in population of the *Anopheles gambiae* complex mosquitoes across the location and between the type A and type B wing (Table 2).

**Table 2:** Variation in wing types among *Anopheles gambiae* complex collected in the study area.

Location	No positive for *An. gambiae* complex	Wing type A		Wing type B		Wing A and B Chi-square (sig.)	Degree of freedom (df)
		*An. gambiae* s. s (%)	*An. arabiensis *(%)	*An. gambiae* s. s (%)	*An. arabiensis *(%)		
Igoba	107 (20.19)	49 (17.31)	0	32 (21.33)	26 (26.80)	374.30 (0.00)	1
Isinigbo	106 (20.00)	53 (18.73)	0	30 (20.00)	23 (23.71)		
Oba–Ile	108 (20.38)	79 (27.92)	0	19 (12.67)	10 (10.31)		
Iju	105 (19.81)	54 (19.08)	0	33 (22.00)	18 (18.56)		
Ita–Ogbolu	104 (19.62)	48 (16.96)	0	36 (24.00)	20 (20.62)		
Total	530	283 (53.39)	0 (0)	150 (28.30)	97 (18.30)		

χ^2^ = 24.39; df = 4; P = 0.02 (P < 0.05).

Wing type A: Wing with a pale interruption proceed with the sub-coastal pale spot.

Wing type B: Wing with a pale interruption found within the pre-apical dark spot.

### Molecular identification of *Anopheles funestus* group

**Figure 3:**
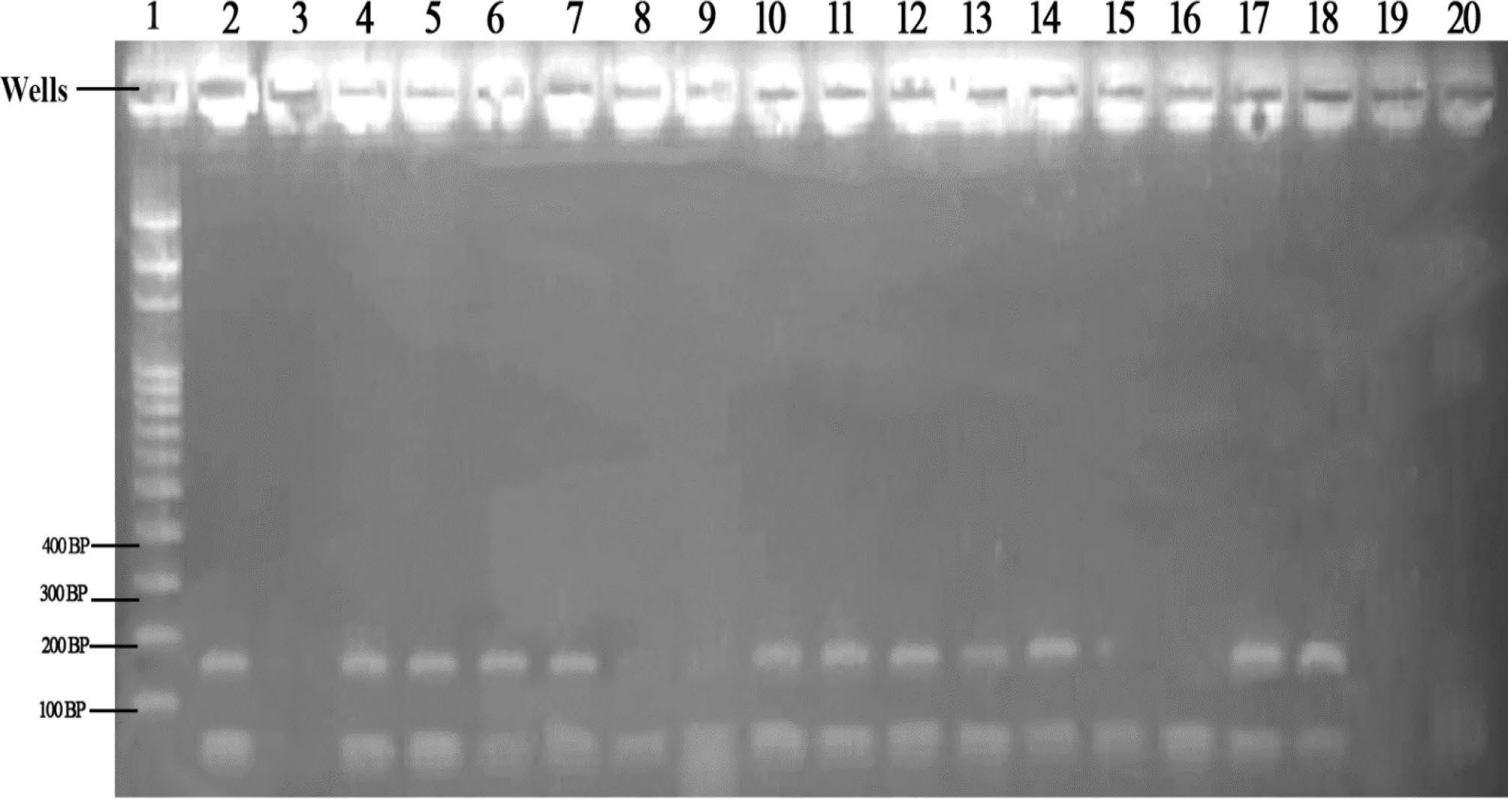
Agarose gel of the DNA fragment of amplified PCR of *Anopheles funestus* group. Well 1 represent 100 bp DNA ladder. Well 2 a positive control, well 3 a negative control, well 4, 5, 6, 7, 10, 11, 12, 13, 14, 17 and 18 represent *Anopheles leesoni* 146 bp.

Among the 200 samples of morphologically identified as belonging to *Anopheles funestus*, 182 (91.0%) of the studied samples were *Anopheles leesoni* (a member of *funestus* group) and 9.0% failed the amplify (Table 3). A representative of agarose gel image showing the DNA fragment bands were presented on Fig. 3. *Anopheles leesoni* was identified on the gel at 146 base pair.

**Table 3:** Molecular identification of *Anopheles funestus* group in the study area.

Location	No examined	*Anopheles leesoni* (%)	Negative (%)
Igoba	40	36 (90.0)	4 (10.0)
Isinigbo	40	37 (92.5)	3 (7.5)
Oba Ile	40	38 (95.0)	2 (5.0)
Iju	40	37 (92.5)	3 (7.5)
Ita–Ogbolu	40	34 (85.0)	6 (15.0)
Total	200	182 (91.0)	18 (9.0)

χ^2^ = 557.72; df = 4; P = 0.01.

## Discussion

The molecular identification of malaria vectors in the study location confirmed three important species of *Anopheles* mosquitoes responsible for malaria transmission in the study area. Two of these malaria vectors belong to the complex of *Anopheles gambiae* (*An. gambiae* s. s. and *An. arabiensis*) while *An. leesoni* was the only member of *An. funestus* group found in the study area. These three species of *Anopheles* mosquitos are major malaria parasite vectors in Afro–tropical countries. According to Rozendaal^15^, the identified species of *Anopheles* mosquitoes in the area are the vector of public health importance due to their anthropophilic behaviour. *An. gambiae* s.s. is the most abundant in the studied locations and the most important within the member of *Anopheles gambiae* complex. Fahmy et al.^16^ and Kabbale et al*.*^17^ have described *An. gambiae* s. s. as the largest anthropophilic among *Anopheles* mosquito species due to their preference for human blood, and they are primarily common around human dwellings. The preference of *An. gambiae* s.s. for human blood and their endophagic behaviour makes this insect the most important vector of malaria parasites in Afro–tropical regions where they are more abundant. The present research work supports the finding of Kabbale et al*.*^17^. In the research carried out by Hamza et al.^18^, *An. arabiensis* was reported to be predominant over *An. gambiae* s.s. but this was different from what was reported in this present study, which described *An. gambiae* s.s. more predominant and widespread compared to *An. arabiensis* in all study locations. *Anopheles gambiae* s.s. and *An. arabiensis* occur sympatrically in the same breeding habitat.

The two forms (A and B) of wings of identified *An. gambiae* s.s. and *An. arabiensis* was presented earlier in Fig. 2a and b. This present study revealed that all the species with the wing type A are molecularly identified as *An. gambiae* s.s., this character could be used in rapid field identification of members of *An. gambiae* complex in the study location. In their study, Sanford et al.^19^ described the importance of wing characters of the two molecular forms of *An. gambiae* s.s. which include the measurement of morphometric characters of the sympatric species distribution from their study areas. The molecular identification of the *An. gambiae* complex mosquito samples with the wing type B revealed two species, which are *An. gambiae* s.s. and *An. arabiensis*. This result shows that using wing type B in sorting out the species member of *An. gambiae* complex might lead to species discrimination. In the study of the wings and molecular identification of these vectors, the result of this present study has shown that *An. gambiae* s.s. can have both types of wings (type A and B). *Anopheles gambiae* with wing type A are more predominant compare to those wing type B in the study locations. The outcome of the present study has revealed that some of the species of *An. gambiae* s.s. and *An. arabiensis* share wing type B. The only method for separating both species is by using the molecular approach.

In this present study, the only member of *An. funestus* group confirmed in the study location was *An. leesoni*. *Anopheles leesoni* has been reported as a zoophilic member of the *An. funestus* group^20^. Among the member of *An. funestus* group, *An. funestus* s.s. is the only member of this group that is anthropophilic and responsible for the transmission of malaria parasites in Africa, other members of this group prefer animal blood. *Anopheles leesoni*, *An. parensis* and *An. rivulorum*, which are primarily zoophilic, have been reported by Temu et al*.*^6^ feeding on human blood (anthropophilic) and found infected with *Plasmodium falciparum* and transmission of the malaria parasites.

All the *An. leesoni* identified in this present study, was found within the human dwellings; this contradicts the earlier report of Gillies and De Meillon^20^, which reported that *An. leesoni* found close to cattle ranches and feeding mainly on cattle. Kamau et al.^21^ reported *An. leesoni* is found close to human dwellings in their study in Kenya. In Nigeria, a West Africa region of Africa, endophilic and anthropophagic behaviour of *An. leesoni* has been reported by Awolola et al.^22^. Kent and Norris^23^ have reported a collection of *An. leesoni* already fed on either goat or cattle around human dwellings in Zambia; this suggested that *An. leesoni* have the tendency of feeding on both human (anthropophilic) and animal (zoophilic) blood and also feed indoor (endophagic) and outdoor (exophagic).

## Conclusions

The molecular identification has revealed that *Anopheles gambiae* s.s. can have different type of wing (A and B). Further study is needed to assess whether individual with different types of wings have different competency in malaria transmission. Presence of *An. leesoni* in the study area shows that the vector could also contribute to the burden malaria infection in the study locations, however, further exploration is needed toward this end.

## Data Availability

All analysed data involved in this study are included in this manuscript.

## Abbreviations

DNA: Deoxyribonucleic acid (DNA)
PCR: Polymerase chain reaction
ddH_2_O: Distillated water
µl: Microlitre
AR: *Anopheles arabiensis* Primer
GA: *Anopheles gambiae* S. s. primer
QD: *Anopheles quadriannulatus* Primer
ME: *Anopheles merus* Primer
VAN: *Anopheles vaneedeni* Primer
PAR: *Anopheles parensis* Primer
LEES: *Anopheles leesoni* Primer
TBE: Tris-borate ethylene-di-amino tetra acid
EtBr: Ethidium bromide
BP: Base pair
